# *cis*-Bis[(4-nitro­phen­yl)cyanamido-κ*N* ^1^]bis­(1,10-phenanthroline-κ^2^ *N*,*N*′)nickel(II) methanol monosolvate

**DOI:** 10.1107/S1600536812009890

**Published:** 2012-03-14

**Authors:** Hossein Chiniforoshan, Mehdi Jazestani, Behrouz Notash

**Affiliations:** a Department of Chemistry, Isfahan University of Technology, Isfahan 84456-38111, Iran; b Department of Chemistry, Shahid Beheshti University, G. C., Evin, Tehran 1983963113, Iran

## Abstract

In the title compound, [Ni(C_7_H_4_N_3_O_2_)_2_(C_12_H_8_N_2_)_2_]·CH_3_OH, the Ni^II^ atom is six-coordinated in a distorted N_6_ octa­hedral geometry and is chelated by two phenanthroline ligands and two phenyl­cyanamide groups which occupy *cis* positions. The (4-nitro­phen­yl)cyanamide anions act as monodentate ligands. There is one classical inter­molecular O—H⋯N hydrogen bond and several C—H⋯O hydrogen bonds are also observed.

## Related literature

For background to phenyl­cyanamide ligands and their complexes, see: Crutchley (2001 ▶). For mononuclear complexes of phenyl­cyanamide complexes, see: Letcher *et al.* (1993 ▶); Kim *et al.* (2002 ▶); Shen *et al.* (1999 ▶). For polynuclear complexes of phenyl­cyanamide ligands, see: Ainscough *et al.* (1991 ▶); Chiniforoshan *et al.* (2009 ▶, 2010 ▶, 2012 ▶); Escuer *et al.* (2004 ▶). For related structures, see: Wu *et al.* (2004 ▶); Cheng *et al.* (2002 ▶); Shen *et al.* (1999 ▶). For the preparation of 4-nitro-phenyl­cyanamide used in the synthesis of the title compound, see: Crutchley & Naklicki (1989 ▶).
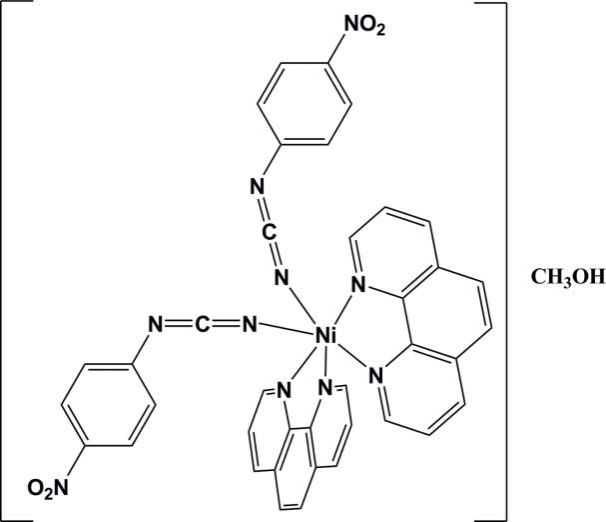


## Experimental

### 

#### Crystal data

[Ni(C_7_H_4_N_3_O_2_)_2_(C_12_H_8_N_2_)_2_]·CH_4_O
*M* *_r_* = 775.40
Triclinic, 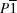

*a* = 10.019 (2) Å
*b* = 11.307 (2) Å
*c* = 16.403 (3) Å
α = 103.54 (3)°
β = 92.96 (3)°
γ = 99.77 (3)°
*V* = 1772.3 (7) Å^3^
*Z* = 2
Mo *K*α radiation
μ = 0.61 mm^−1^
*T* = 298 K
0.25 × 0.20 × 0.10 mm

#### Data collection

Stoe IPDS II diffractometer
Absorption correction: numerical (*X-RED* and *X-SHAPE*; Stoe & Cie, 2005 ▶) *T*_min_ = 0.862, *T*_max_ = 0.938
19823 measured reflections
9512 independent reflections
6693 reflections with *I* > 2σ(*I*)
*R*_int_ = 0.053

#### Refinement

*R*[*F* ^2^ > 2σ(*F* ^2^)] = 0.061
*wR*(*F* ^2^) = 0.139
*S* = 1.06
9512 reflections
501 parameters
1 restraint
H atoms treated by a mixture of independent and constrained refinement
Δρ_max_ = 0.65 e Å^−3^
Δρ_min_ = −0.31 e Å^−3^

### 

Data collection: *X-AREA* (Stoe & Cie, 2005 ▶); cell refinement: *X-AREA*; data reduction: *X-AREA*; program(s) used to solve structure: *SHELXS97* (Sheldrick, 2008 ▶); program(s) used to refine structure: *SHELXL97* (Sheldrick, 2008 ▶); molecular graphics: *ORTEP-3 for Windows* (Farrugia, 1997 ▶); software used to prepare material for publication: *WinGX* (Farrugia, 1999 ▶).

## Supplementary Material

Crystal structure: contains datablock(s) I, global. DOI: 10.1107/S1600536812009890/bt5812sup1.cif

Structure factors: contains datablock(s) I. DOI: 10.1107/S1600536812009890/bt5812Isup2.hkl

Additional supplementary materials:  crystallographic information; 3D view; checkCIF report

## Figures and Tables

**Table 1:** Hydrogen-bond geometry (Å, °)

*D*—H⋯*A*	*D*—H	H⋯*A*	*D*⋯*A*	*D*—H⋯*A*
O5—H5*A*⋯N9^i^	0.91 (3)	1.98 (4)	2.883 (4)	174 (5)
C39—H39*B*⋯O4^ii^	0.96	2.55	3.435 (7)	153
C22—H22⋯O1^iii^	0.93	2.57	3.469 (6)	162
C16—H16⋯O2^iv^	0.93	2.45	3.312 (6)	154

Symmetry codes: (i) 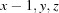; (ii) 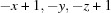; (iii) 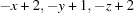; (iv) 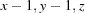.

**Fig. 1.**
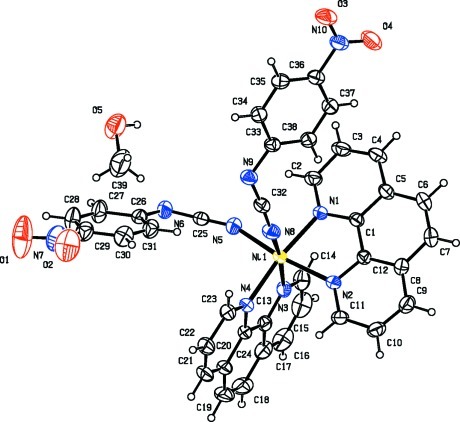
The asymmetric unit of the title compound with displacement ellipsoids drawn at 30% probability level.

**Fig. 2.**
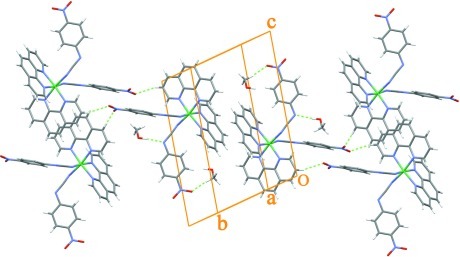
The packing diagram of [Ni(Phen)2(4-NO2-pcyd)2].CH3OH. The intermolecular O—H···N and C—H···O hydrogen bonds are shown as green dashed lines.

## supplementary crystallographic information

### Comment

Phenylcyanmide ligands (pcyd) can act as monodentate (Letcher *et al.*, 1993; Kim *et al.*, 2002; Shen *et al.*, 1999) and also as bridging ligands (Crutchley, 2001). In the bridging mode, the cyanamido group (NCN) is coordinated in the end-to-end mode, forming polynuclear complexes (Chiniforoshan *et al.* 2009, 2010, 2012; Escuer *et al.*, 2004; Ainscough *et al.*, 1991).

Following our work with this family of ligands, we report here the synthesis and crystal structure of mononuclear [Ni(Phen)_2_(4-NO_2_-pcyd)_2_].CH_3_OH compound of (4-nitrophenyl)cyanamide ligand, Crutchley & Naklicki (1989). The asymmetric unit of the title compound is shown in Fig. 1. In the structure of the title compound, nickel(II) atom has a distorted octahedral geometry (Fig. 1). The coordination environment consist of four nitrogen atoms from two 1,10-phenanthroline ligand and two anionic 4-NO_2_-phenylcyanamide ligands which occupy *cis* position. Bond lengths and angles are in the normal ranges reported for similar structures (Wu *et al.*, 2004; Cheng *et al.*, 2002; Shen *et al.*, 1999). Crutchley (2001) has shown that the angle of a metal atom with the axial CN moiety ranges from 180° to 120°. These angles for the title compound are equal to 153.2 (2) and 150.6 (3)° for Ni(1)—N(5)—C(25) and Ni(1)—N(8)—C(32), respectively. There are several intermolecular O—H···N and C—H···O hydrogen bonds which play important role in the stabilization of crystal structure (Table 1 & Fig. 2).

### Experimental

A solution of Ni(OAc)_2_.4H_2_O (0.24 gr, 0.1 mmol) in 25 ml of methanol was slowly added to methanolic solution (in 35 ml) of 4-nitrophenylcynamide (Crutchley & Naklicki, 1989) (0.32 gr, 0.2 mmol) and 1,10-phenanthroline (0.39 gr, 0.2 mmol). The mixture was stirred at ambient temperature and the yellow solid filtered after 5 h. The yellow crystals suitable for X-ray structure determination were obtained by dissolving this solid in DMF then diffused by methanol after 3 weeks.

### Refinement

The hydrogen atom attached to oxygen atom of the methanol was found in difference Fourier map and refined isotropically with distance restraint of O—H = 0.91 (3)Å. All H atoms bonded to C were positioned geometrically and refined as riding atoms with C—H = 0.93 Å and *U*iso(H) = 1.2 *U*eq(C) for aromatic C, C—H = 0.96 Å and *U*iso(H) = 1.5 *Ueq*(C) for methyl groups.

### Crystal data

**Table:**

[Ni(C_7_H_4_N_3_O_2_)_2_(C_12_H_8_N_2_)_2_]·CH_4_O	*Z* = 2
*M**_r_* = 775.40	*F*(000) = 800
Triclinic, *P*1	*D*_x_ = 1.453 Mg m^−^^3^
Hall symbol: -P 1	Mo *K*α radiation, λ = 0.71073 Å
*a* = 10.019 (2) Å	Cell parameters from 9512 reflections
*b* = 11.307 (2) Å	θ = 2.1–29.2°
*c* = 16.403 (3) Å	µ = 0.61 mm^−^^1^
α = 103.54 (3)°	*T* = 298 K
β = 92.96 (3)°	Plate, yellow
γ = 99.77 (3)°	0.25 × 0.2 × 0.1 mm
*V* = 1772.3 (7) Å^3^	

### Data collection

**Table:**

Stoe IPDS II diffractometer	9512 independent reflections
Radiation source: fine-focus sealed tube	6693 reflections with *I* > 2σ(*I*)
Graphite monochromator	*R*_int_ = 0.053
Detector resolution: 0.15 mm pixels mm^-1^	θ_max_ = 29.2°, θ_min_ = 2.1°
rotation method scans	*h* = −12→13
Absorption correction: numerical (*X-RED* and *X-SHAPE*; Stoe & Cie, 2005)	*k* = −15→15
*T*_min_ = 0.862, *T*_max_ = 0.938	*l* = −22→20
19823 measured reflections	

### Refinement

**Table:**

Refinement on *F*^2^	Primary atom site location: structure-invariant direct methods
Least-squares matrix: full	Secondary atom site location: difference Fourier map
*R*[*F*^2^ > 2σ(*F*^2^)] = 0.061	Hydrogen site location: inferred from neighbouring sites
*wR*(*F*^2^) = 0.139	H atoms treated by a mixture of independent and constrained refinement
*S* = 1.06	*w* = 1/[σ^2^(*F*_o_^2^) + (0.0512*P*)^2^ + 0.8996*P*] where *P* = (*F*_o_^2^ + 2*F*_c_^2^)/3
9512 reflections	(Δ/σ)_max_ = 0.001
501 parameters	Δρ_max_ = 0.65 e Å^−^^3^
1 restraint	Δρ_min_ = −0.31 e Å^−^^3^

### Special details

**Table:**

Experimental. shape of crystal determined optically
Geometry. All e.s.d.'s (except the e.s.d. in the dihedral angle between two l.s. planes) are estimated using the full covariance matrix. The cell e.s.d.'s are taken into account individually in the estimation of e.s.d.'s in distances, angles and torsion angles; correlations between e.s.d.'s in cell parameters are only used when they are defined by crystal symmetry. An approximate (isotropic) treatment of cell e.s.d.'s is used for estimating e.s.d.'s involving l.s. planes.
Refinement. Refinement of *F*^2^ against ALL reflections. The weighted *R*-factor *wR* and goodness of fit *S* are based on *F*^2^, conventional *R*-factors *R* are based on *F*, with *F* set to zero for negative *F*^2^. The threshold expression of *F*^2^ > σ(*F*^2^) is used only for calculating *R*-factors(gt) *etc*. and is not relevant to the choice of reflections for refinement. *R*-factors based on *F*^2^ are statistically about twice as large as those based on *F*, and *R*- factors based on ALL data will be even larger.

### Fractional atomic coordinates and isotropic or equivalent isotropic displacement parameters (Å^2^)

**Table:**

	*x*	*y*	*z*	*U*_iso_*/*U*_eq_	
C39	0.1500 (7)	0.3400 (5)	0.7660 (5)	0.146 (3)	
H39A	0.2378	0.3493	0.7450	0.219*	
H39B	0.1205	0.2554	0.7678	0.219*	
H39C	0.1557	0.3928	0.8217	0.219*	
O1	0.8551 (5)	0.7156 (4)	1.0013 (4)	0.197 (3)	
O2	1.0049 (4)	0.6144 (4)	0.9548 (3)	0.1357 (15)	
Ni1	0.62348 (4)	−0.11112 (3)	0.714358 (19)	0.04018 (11)	
N2	0.7128 (2)	−0.2733 (2)	0.68911 (13)	0.0435 (5)	
N3	0.4682 (2)	−0.2034 (2)	0.77044 (14)	0.0484 (5)	
N1	0.5513 (2)	−0.1913 (2)	0.58779 (13)	0.0449 (5)	
N4	0.7020 (2)	−0.0547 (2)	0.83983 (13)	0.0445 (5)	
N8	0.7899 (3)	−0.0105 (3)	0.67656 (16)	0.0583 (6)	
N5	0.5203 (3)	0.0329 (2)	0.72202 (17)	0.0590 (6)	
C12	0.6933 (3)	−0.3347 (2)	0.60642 (15)	0.0418 (5)	
C5	0.5907 (3)	−0.3441 (3)	0.46523 (17)	0.0538 (7)	
C24	0.6222 (3)	−0.1045 (3)	0.89187 (16)	0.0469 (6)	
C13	0.4963 (3)	−0.1838 (3)	0.85447 (17)	0.0490 (6)	
C11	0.7872 (3)	−0.3163 (3)	0.74050 (18)	0.0532 (7)	
H11	0.8019	−0.2746	0.7972	0.064*	
C20	0.6575 (4)	−0.0808 (3)	0.97889 (18)	0.0628 (9)	
C6	0.6549 (4)	−0.4473 (3)	0.4333 (2)	0.0713 (10)	
H6	0.6451	−0.4833	0.3757	0.086*	
C1	0.6097 (3)	−0.2884 (2)	0.55207 (15)	0.0438 (6)	
N9	0.8915 (3)	0.1479 (2)	0.60677 (15)	0.0534 (6)	
C38	0.8241 (3)	0.0062 (3)	0.46698 (18)	0.0505 (6)	
H38	0.7834	−0.0565	0.4906	0.061*	
C22	0.8588 (4)	0.0513 (3)	0.9591 (2)	0.0735 (10)	
H22	0.9395	0.1066	0.9805	0.088*	
C33	0.8870 (3)	0.1206 (3)	0.51983 (17)	0.0460 (6)	
C8	0.7502 (3)	−0.4389 (3)	0.57381 (19)	0.0540 (7)	
C14	0.3505 (3)	−0.2741 (3)	0.7350 (2)	0.0651 (9)	
H14	0.3292	−0.2865	0.6774	0.078*	
C32	0.8361 (3)	0.0614 (3)	0.64100 (17)	0.0486 (6)	
C36	0.8833 (3)	0.0780 (3)	0.34610 (17)	0.0502 (6)	
C35	0.9464 (3)	0.1919 (3)	0.39645 (19)	0.0571 (7)	
H35	0.9871	0.2539	0.3722	0.069*	
C34	0.9486 (3)	0.2129 (3)	0.48241 (19)	0.0546 (7)	
H34	0.9915	0.2893	0.5163	0.065*	
C37	0.8216 (3)	−0.0150 (3)	0.38064 (18)	0.0532 (7)	
H37	0.7790	−0.0911	0.3461	0.064*	
C23	0.8176 (3)	0.0209 (3)	0.87307 (19)	0.0561 (7)	
H23	0.8735	0.0551	0.8377	0.067*	
C2	0.4720 (3)	−0.1491 (3)	0.53848 (19)	0.0578 (7)	
H2	0.4306	−0.0834	0.5627	0.069*	
C3	0.4480 (4)	−0.1994 (3)	0.4516 (2)	0.0665 (9)	
H3	0.3917	−0.1674	0.4189	0.080*	
C19	0.5667 (6)	−0.1375 (4)	1.0287 (2)	0.0860 (13)	
H19	0.5901	−0.1234	1.0862	0.103*	
C9	0.8266 (4)	−0.4821 (3)	0.6308 (2)	0.0634 (8)	
H9	0.8647	−0.5520	0.6121	0.076*	
C21	0.7808 (5)	0.0001 (4)	1.0112 (2)	0.0774 (11)	
H21	0.8089	0.0185	1.0685	0.093*	
C17	0.4077 (4)	−0.2368 (3)	0.9054 (2)	0.0660 (9)	
C10	0.8445 (4)	−0.4207 (3)	0.7137 (2)	0.0625 (8)	
H10	0.8948	−0.4486	0.7522	0.075*	
O3	0.9376 (3)	0.1389 (3)	0.22532 (15)	0.0832 (8)	
N10	0.8849 (3)	0.0551 (3)	0.25539 (16)	0.0626 (7)	
O4	0.8363 (4)	−0.0475 (3)	0.21191 (16)	0.1014 (10)	
C4	0.5077 (4)	−0.2955 (3)	0.41506 (18)	0.0659 (9)	
H4	0.4936	−0.3287	0.3571	0.079*	
C7	0.7291 (4)	−0.4929 (3)	0.4848 (2)	0.0706 (9)	
H7	0.7677	−0.5612	0.4622	0.085*	
C18	0.4481 (6)	−0.2108 (4)	0.9942 (3)	0.0871 (14)	
H18	0.3906	−0.2458	1.0285	0.104*	
C15	0.2576 (4)	−0.3304 (4)	0.7812 (4)	0.0866 (13)	
H15	0.1766	−0.3807	0.7544	0.104*	
C16	0.2862 (4)	−0.3115 (4)	0.8657 (3)	0.0862 (13)	
H16	0.2244	−0.3485	0.8970	0.103*	
N6	0.4741 (3)	0.2393 (2)	0.78229 (17)	0.0612 (7)	
C25	0.5024 (3)	0.1317 (3)	0.75123 (17)	0.0502 (7)	
C26	0.5794 (3)	0.3316 (3)	0.82375 (18)	0.0535 (7)	
C31	0.7151 (4)	0.3199 (3)	0.8274 (2)	0.0640 (8)	
H31	0.7387	0.2463	0.7986	0.077*	
C30	0.8150 (4)	0.4140 (3)	0.8722 (3)	0.0731 (10)	
H30	0.9053	0.4043	0.8742	0.088*	
C29	0.7803 (4)	0.5236 (3)	0.9144 (2)	0.0706 (9)	
C27	0.5480 (4)	0.4449 (3)	0.8667 (3)	0.0801 (11)	
H27	0.4581	0.4561	0.8646	0.096*	
N7	0.8870 (5)	0.6246 (4)	0.9598 (3)	0.1013 (12)	
C28	0.6476 (5)	0.5385 (4)	0.9115 (3)	0.0856 (12)	
H28	0.6253	0.6127	0.9401	0.103*	
O5	0.0518 (4)	0.3745 (3)	0.7102 (3)	0.1121 (12)	
H5A	0.001 (5)	0.301 (4)	0.681 (3)	0.14 (2)*	

### Atomic displacement parameters (Å^2^)

**Table:**

	*U*^11^	*U*^22^	*U*^33^	*U*^12^	*U*^13^	*U*^23^
C39	0.175 (6)	0.078 (3)	0.169 (6)	0.031 (4)	−0.033 (5)	0.007 (4)
O1	0.132 (4)	0.093 (3)	0.301 (7)	0.003 (3)	−0.012 (4)	−0.062 (4)
O2	0.088 (2)	0.105 (3)	0.196 (4)	−0.015 (2)	0.031 (3)	0.020 (3)
Ni1	0.0489 (2)	0.04276 (18)	0.02925 (15)	0.01033 (14)	0.00388 (12)	0.00828 (12)
N2	0.0498 (13)	0.0472 (12)	0.0352 (10)	0.0105 (10)	0.0052 (9)	0.0125 (9)
N3	0.0533 (14)	0.0422 (12)	0.0478 (12)	0.0082 (10)	0.0090 (10)	0.0071 (10)
N1	0.0528 (13)	0.0485 (12)	0.0353 (10)	0.0150 (10)	0.0015 (9)	0.0106 (9)
N4	0.0549 (13)	0.0440 (12)	0.0337 (10)	0.0103 (10)	0.0015 (9)	0.0075 (9)
N8	0.0629 (16)	0.0673 (16)	0.0467 (13)	0.0077 (13)	0.0109 (11)	0.0199 (12)
N5	0.0680 (17)	0.0530 (15)	0.0569 (15)	0.0198 (13)	0.0024 (12)	0.0099 (12)
C12	0.0457 (14)	0.0413 (13)	0.0382 (12)	0.0080 (11)	0.0069 (10)	0.0091 (10)
C5	0.0667 (19)	0.0549 (17)	0.0357 (13)	0.0074 (14)	0.0038 (12)	0.0061 (12)
C24	0.0654 (18)	0.0447 (14)	0.0349 (12)	0.0209 (13)	0.0090 (12)	0.0097 (10)
C13	0.0628 (18)	0.0446 (14)	0.0460 (14)	0.0204 (13)	0.0207 (13)	0.0131 (11)
C11	0.0613 (18)	0.0604 (18)	0.0442 (14)	0.0168 (14)	0.0043 (13)	0.0219 (13)
C20	0.097 (3)	0.0649 (19)	0.0360 (14)	0.0398 (19)	0.0114 (15)	0.0131 (13)
C6	0.100 (3)	0.066 (2)	0.0414 (15)	0.0221 (19)	0.0067 (16)	−0.0039 (14)
C1	0.0502 (15)	0.0450 (14)	0.0347 (12)	0.0050 (11)	0.0051 (10)	0.0092 (10)
N9	0.0590 (15)	0.0565 (14)	0.0452 (12)	0.0045 (12)	0.0082 (11)	0.0176 (11)
C38	0.0542 (16)	0.0490 (15)	0.0496 (15)	0.0060 (13)	0.0079 (12)	0.0168 (12)
C22	0.089 (3)	0.066 (2)	0.0548 (19)	0.0179 (19)	−0.0261 (18)	−0.0005 (16)
C33	0.0447 (14)	0.0527 (15)	0.0444 (13)	0.0121 (12)	0.0077 (11)	0.0163 (12)
C8	0.0627 (18)	0.0445 (15)	0.0544 (16)	0.0136 (13)	0.0079 (14)	0.0086 (12)
C14	0.0533 (18)	0.0570 (19)	0.078 (2)	0.0074 (15)	0.0052 (16)	0.0051 (16)
C32	0.0472 (15)	0.0584 (17)	0.0404 (13)	0.0120 (13)	0.0062 (11)	0.0110 (12)
C36	0.0489 (16)	0.0638 (18)	0.0424 (14)	0.0161 (13)	0.0069 (12)	0.0175 (13)
C35	0.0649 (19)	0.0590 (18)	0.0526 (16)	0.0087 (15)	0.0142 (14)	0.0240 (14)
C34	0.0625 (18)	0.0491 (16)	0.0509 (15)	0.0034 (14)	0.0116 (13)	0.0135 (13)
C37	0.0540 (17)	0.0546 (17)	0.0494 (15)	0.0075 (13)	0.0041 (13)	0.0115 (13)
C23	0.0627 (18)	0.0497 (16)	0.0507 (16)	0.0066 (14)	−0.0062 (13)	0.0072 (13)
C2	0.0635 (19)	0.069 (2)	0.0455 (15)	0.0245 (15)	−0.0018 (13)	0.0161 (14)
C3	0.075 (2)	0.083 (2)	0.0442 (16)	0.0182 (18)	−0.0085 (15)	0.0212 (16)
C19	0.132 (4)	0.105 (3)	0.0445 (18)	0.059 (3)	0.033 (2)	0.033 (2)
C9	0.074 (2)	0.0528 (18)	0.071 (2)	0.0258 (16)	0.0141 (17)	0.0182 (15)
C21	0.114 (3)	0.082 (2)	0.0356 (15)	0.042 (2)	−0.0144 (18)	0.0012 (16)
C17	0.077 (2)	0.0609 (19)	0.077 (2)	0.0283 (18)	0.0401 (18)	0.0321 (17)
C10	0.070 (2)	0.064 (2)	0.0648 (19)	0.0242 (16)	0.0056 (16)	0.0302 (16)
O3	0.110 (2)	0.0929 (19)	0.0529 (13)	0.0118 (16)	0.0165 (13)	0.0331 (13)
N10	0.0711 (18)	0.0763 (19)	0.0446 (13)	0.0176 (15)	0.0061 (12)	0.0204 (13)
O4	0.153 (3)	0.088 (2)	0.0470 (13)	−0.0056 (19)	0.0008 (16)	0.0078 (13)
C4	0.084 (2)	0.077 (2)	0.0333 (13)	0.0120 (19)	−0.0025 (14)	0.0111 (14)
C7	0.095 (3)	0.0550 (19)	0.0598 (19)	0.0275 (18)	0.0138 (18)	−0.0008 (15)
C18	0.123 (4)	0.099 (3)	0.073 (2)	0.057 (3)	0.061 (3)	0.052 (2)
C15	0.053 (2)	0.069 (2)	0.133 (4)	0.0011 (17)	0.020 (2)	0.020 (2)
C16	0.076 (3)	0.074 (2)	0.124 (4)	0.019 (2)	0.053 (3)	0.043 (3)
N6	0.0699 (17)	0.0549 (15)	0.0593 (15)	0.0258 (13)	0.0045 (13)	0.0055 (12)
C25	0.0619 (18)	0.0566 (17)	0.0366 (13)	0.0188 (14)	0.0046 (12)	0.0151 (12)
C26	0.071 (2)	0.0543 (17)	0.0446 (14)	0.0262 (15)	0.0147 (13)	0.0174 (12)
C31	0.074 (2)	0.0528 (18)	0.071 (2)	0.0186 (16)	0.0279 (17)	0.0172 (15)
C30	0.065 (2)	0.071 (2)	0.088 (3)	0.0138 (18)	0.0259 (19)	0.024 (2)
C29	0.079 (2)	0.0532 (19)	0.080 (2)	0.0059 (17)	0.0168 (19)	0.0199 (17)
C27	0.077 (2)	0.063 (2)	0.097 (3)	0.0332 (19)	0.002 (2)	−0.0019 (19)
N7	0.096 (3)	0.066 (2)	0.135 (4)	0.005 (2)	0.016 (3)	0.018 (2)
C28	0.091 (3)	0.057 (2)	0.104 (3)	0.029 (2)	0.012 (2)	−0.001 (2)
O5	0.112 (3)	0.070 (2)	0.144 (3)	0.0153 (18)	−0.024 (2)	0.016 (2)

### Geometric parameters (Å, º)

**Table:**

C39—O5	1.471 (7)	C8—C7	1.434 (4)
C39—H39A	0.9600	C14—C15	1.394 (6)
C39—H39B	0.9600	C14—H14	0.9300
C39—H39C	0.9600	C36—C37	1.380 (4)
O1—N7	1.195 (6)	C36—C35	1.383 (4)
O2—N7	1.212 (5)	C36—N10	1.451 (4)
Ni1—N5	2.056 (3)	C35—C34	1.372 (4)
Ni1—N8	2.062 (3)	C35—H35	0.9300
Ni1—N4	2.076 (2)	C34—H34	0.9300
Ni1—N1	2.097 (2)	C37—H37	0.9300
Ni1—N3	2.098 (2)	C23—H23	0.9300
Ni1—N2	2.139 (2)	C2—C3	1.397 (4)
N2—C11	1.323 (3)	C2—H2	0.9300
N2—C12	1.358 (3)	C3—C4	1.360 (5)
N3—C14	1.326 (4)	C3—H3	0.9300
N3—C13	1.351 (4)	C19—C18	1.340 (6)
N1—C2	1.325 (4)	C19—H19	0.9300
N1—C1	1.358 (3)	C9—C10	1.362 (5)
N4—C23	1.322 (4)	C9—H9	0.9300
N4—C24	1.352 (4)	C21—H21	0.9300
N8—C32	1.157 (4)	C17—C16	1.390 (6)
N5—C25	1.156 (4)	C17—C18	1.438 (6)
C12—C8	1.402 (4)	C10—H10	0.9300
C12—C1	1.435 (4)	O3—N10	1.224 (4)
C5—C4	1.402 (5)	N10—O4	1.215 (4)
C5—C1	1.405 (4)	C4—H4	0.9300
C5—C6	1.433 (5)	C7—H7	0.9300
C24—C20	1.404 (4)	C18—H18	0.9300
C24—C13	1.432 (4)	C15—C16	1.360 (7)
C13—C17	1.407 (4)	C15—H15	0.9300
C11—C10	1.390 (4)	C16—H16	0.9300
C11—H11	0.9300	N6—C25	1.289 (4)
C20—C21	1.403 (6)	N6—C26	1.373 (4)
C20—C19	1.425 (6)	C26—C31	1.388 (5)
C6—C7	1.342 (5)	C26—C27	1.406 (4)
C6—H6	0.9300	C31—C30	1.368 (5)
N9—C32	1.297 (4)	C31—H31	0.9300
N9—C33	1.383 (3)	C30—C29	1.380 (5)
C38—C37	1.378 (4)	C30—H30	0.9300
C38—C33	1.402 (4)	C29—C28	1.368 (6)
C38—H38	0.9300	C29—N7	1.451 (5)
C22—C21	1.350 (6)	C27—C28	1.363 (6)
C22—C23	1.395 (4)	C27—H27	0.9300
C22—H22	0.9300	C28—H28	0.9300
C33—C34	1.402 (4)	O5—H5A	0.91 (3)
C8—C9	1.401 (5)		
			
O5—C39—H39A	109.5	C37—C36—N10	119.4 (3)
O5—C39—H39B	109.5	C35—C36—N10	119.3 (3)
H39A—C39—H39B	109.5	C34—C35—C36	119.5 (3)
O5—C39—H39C	109.5	C34—C35—H35	120.2
H39A—C39—H39C	109.5	C36—C35—H35	120.2
H39B—C39—H39C	109.5	C35—C34—C33	120.9 (3)
N5—Ni1—N8	90.67 (12)	C35—C34—H34	119.6
N5—Ni1—N4	94.32 (10)	C33—C34—H34	119.6
N8—Ni1—N4	92.39 (10)	C38—C37—C36	119.1 (3)
N5—Ni1—N1	93.16 (10)	C38—C37—H37	120.4
N8—Ni1—N1	89.69 (10)	C36—C37—H37	120.4
N4—Ni1—N1	172.22 (9)	N4—C23—C22	122.5 (3)
N5—Ni1—N3	89.86 (11)	N4—C23—H23	118.7
N8—Ni1—N3	171.74 (10)	C22—C23—H23	118.7
N4—Ni1—N3	79.35 (10)	N1—C2—C3	122.6 (3)
N1—Ni1—N3	98.51 (10)	N1—C2—H2	118.7
N5—Ni1—N2	171.53 (9)	C3—C2—H2	118.7
N8—Ni1—N2	91.08 (10)	C4—C3—C2	119.5 (3)
N4—Ni1—N2	93.89 (9)	C4—C3—H3	120.3
N1—Ni1—N2	78.56 (9)	C2—C3—H3	120.3
N3—Ni1—N2	89.59 (9)	C18—C19—C20	121.2 (3)
C11—N2—C12	117.8 (2)	C18—C19—H19	119.4
C11—N2—Ni1	129.65 (19)	C20—C19—H19	119.4
C12—N2—Ni1	112.50 (17)	C10—C9—C8	119.4 (3)
C14—N3—C13	118.2 (3)	C10—C9—H9	120.3
C14—N3—Ni1	129.1 (2)	C8—C9—H9	120.3
C13—N3—Ni1	112.70 (19)	C22—C21—C20	119.9 (3)
C2—N1—C1	118.3 (2)	C22—C21—H21	120.1
C2—N1—Ni1	127.8 (2)	C20—C21—H21	120.1
C1—N1—Ni1	113.33 (17)	C16—C17—C13	117.1 (4)
C23—N4—C24	118.1 (2)	C16—C17—C18	124.5 (4)
C23—N4—Ni1	128.3 (2)	C13—C17—C18	118.4 (4)
C24—N4—Ni1	113.61 (18)	C9—C10—C11	119.6 (3)
C32—N8—Ni1	150.6 (3)	C9—C10—H10	120.2
C25—N5—Ni1	153.2 (2)	C11—C10—H10	120.2
N2—C12—C8	122.8 (3)	O4—N10—O3	122.2 (3)
N2—C12—C1	116.9 (2)	O4—N10—C36	118.9 (3)
C8—C12—C1	120.2 (2)	O3—N10—C36	118.9 (3)
C4—C5—C1	117.4 (3)	C3—C4—C5	119.6 (3)
C4—C5—C6	124.0 (3)	C3—C4—H4	120.2
C1—C5—C6	118.6 (3)	C5—C4—H4	120.2
N4—C24—C20	123.0 (3)	C6—C7—C8	121.6 (3)
N4—C24—C13	116.9 (2)	C6—C7—H7	119.2
C20—C24—C13	120.1 (3)	C8—C7—H7	119.2
N3—C13—C17	122.9 (3)	C19—C18—C17	121.6 (3)
N3—C13—C24	117.4 (2)	C19—C18—H18	119.2
C17—C13—C24	119.7 (3)	C17—C18—H18	119.2
N2—C11—C10	123.1 (3)	C16—C15—C14	119.6 (4)
N2—C11—H11	118.5	C16—C15—H15	120.2
C10—C11—H11	118.5	C14—C15—H15	120.2
C21—C20—C24	116.8 (3)	C15—C16—C17	119.9 (4)
C21—C20—C19	124.3 (3)	C15—C16—H16	120.0
C24—C20—C19	118.9 (4)	C17—C16—H16	120.0
C7—C6—C5	121.4 (3)	C25—N6—C26	117.3 (3)
C7—C6—H6	119.3	N5—C25—N6	176.2 (4)
C5—C6—H6	119.3	N6—C26—C31	124.4 (3)
N1—C1—C5	122.5 (3)	N6—C26—C27	118.0 (3)
N1—C1—C12	117.7 (2)	C31—C26—C27	117.6 (3)
C5—C1—C12	119.8 (3)	C30—C31—C26	121.5 (3)
C32—N9—C33	117.8 (2)	C30—C31—H31	119.2
C37—C38—C33	121.1 (3)	C26—C31—H31	119.2
C37—C38—H38	119.4	C31—C30—C29	119.3 (4)
C33—C38—H38	119.4	C31—C30—H30	120.3
C21—C22—C23	119.7 (3)	C29—C30—H30	120.3
C21—C22—H22	120.2	C28—C29—C30	120.6 (4)
C23—C22—H22	120.2	C28—C29—N7	120.2 (4)
N9—C33—C38	123.8 (3)	C30—C29—N7	119.2 (4)
N9—C33—C34	118.1 (3)	C28—C27—C26	120.7 (4)
C38—C33—C34	118.1 (3)	C28—C27—H27	119.6
C9—C8—C12	117.4 (3)	C26—C27—H27	119.6
C9—C8—C7	124.3 (3)	O1—N7—O2	122.1 (5)
C12—C8—C7	118.3 (3)	O1—N7—C29	118.6 (5)
N3—C14—C15	122.3 (4)	O2—N7—C29	119.4 (4)
N3—C14—H14	118.9	C27—C28—C29	120.2 (3)
C15—C14—H14	118.9	C27—C28—H28	119.9
N8—C32—N9	175.6 (3)	C29—C28—H28	119.9
C37—C36—C35	121.2 (3)	C39—O5—H5A	104 (4)
			
N8—Ni1—N2—C11	94.1 (3)	N2—C12—C1—C5	177.1 (3)
N4—Ni1—N2—C11	1.6 (3)	C8—C12—C1—C5	−3.2 (4)
N1—Ni1—N2—C11	−176.5 (3)	C32—N9—C33—C38	1.2 (4)
N3—Ni1—N2—C11	−77.7 (3)	C32—N9—C33—C34	−178.9 (3)
N8—Ni1—N2—C12	−82.19 (19)	C37—C38—C33—N9	179.3 (3)
N4—Ni1—N2—C12	−174.66 (18)	C37—C38—C33—C34	−0.6 (4)
N1—Ni1—N2—C12	7.27 (18)	N2—C12—C8—C9	2.0 (4)
N3—Ni1—N2—C12	106.04 (19)	C1—C12—C8—C9	−177.7 (3)
N5—Ni1—N3—C14	83.3 (3)	N2—C12—C8—C7	−176.8 (3)
N4—Ni1—N3—C14	177.7 (3)	C1—C12—C8—C7	3.5 (4)
N1—Ni1—N3—C14	−9.9 (3)	C13—N3—C14—C15	−1.6 (5)
N2—Ni1—N3—C14	−88.2 (3)	Ni1—N3—C14—C15	179.4 (3)
N5—Ni1—N3—C13	−95.8 (2)	C37—C36—C35—C34	0.3 (5)
N4—Ni1—N3—C13	−1.39 (19)	N10—C36—C35—C34	−178.1 (3)
N1—Ni1—N3—C13	171.02 (19)	C36—C35—C34—C33	−0.4 (5)
N2—Ni1—N3—C13	92.6 (2)	N9—C33—C34—C35	−179.4 (3)
N5—Ni1—N1—C2	2.2 (3)	C38—C33—C34—C35	0.5 (5)
N8—Ni1—N1—C2	−88.5 (3)	C33—C38—C37—C36	0.6 (5)
N3—Ni1—N1—C2	92.5 (3)	C35—C36—C37—C38	−0.4 (5)
N2—Ni1—N1—C2	−179.6 (3)	N10—C36—C37—C38	178.0 (3)
N5—Ni1—N1—C1	172.90 (19)	C24—N4—C23—C22	−0.4 (4)
N8—Ni1—N1—C1	82.2 (2)	Ni1—N4—C23—C22	−179.9 (2)
N3—Ni1—N1—C1	−96.77 (19)	C21—C22—C23—N4	1.7 (5)
N2—Ni1—N1—C1	−8.92 (18)	C1—N1—C2—C3	−1.0 (5)
N5—Ni1—N4—C23	−89.7 (3)	Ni1—N1—C2—C3	169.4 (3)
N8—Ni1—N4—C23	1.1 (3)	N1—C2—C3—C4	0.0 (5)
N3—Ni1—N4—C23	−178.8 (3)	C21—C20—C19—C18	177.8 (4)
N2—Ni1—N4—C23	92.4 (3)	C24—C20—C19—C18	−1.1 (6)
N5—Ni1—N4—C24	90.8 (2)	C12—C8—C9—C10	−1.3 (5)
N8—Ni1—N4—C24	−178.4 (2)	C7—C8—C9—C10	177.4 (3)
N3—Ni1—N4—C24	1.73 (18)	C23—C22—C21—C20	−1.6 (6)
N2—Ni1—N4—C24	−87.13 (19)	C24—C20—C21—C22	0.2 (5)
N5—Ni1—N8—C32	−33.3 (5)	C19—C20—C21—C22	−178.6 (4)
N4—Ni1—N8—C32	−127.6 (5)	N3—C13—C17—C16	−0.5 (5)
N1—Ni1—N8—C32	59.9 (5)	C24—C13—C17—C16	179.1 (3)
N2—Ni1—N8—C32	138.4 (5)	N3—C13—C17—C18	179.4 (3)
N8—Ni1—N5—C25	−67.7 (6)	C24—C13—C17—C18	−1.0 (4)
N4—Ni1—N5—C25	24.7 (6)	C8—C9—C10—C11	−0.2 (5)
N1—Ni1—N5—C25	−157.5 (6)	N2—C11—C10—C9	1.2 (5)
N3—Ni1—N5—C25	104.0 (6)	C37—C36—N10—O4	−2.7 (5)
C11—N2—C12—C8	−1.1 (4)	C35—C36—N10—O4	175.7 (3)
Ni1—N2—C12—C8	175.6 (2)	C37—C36—N10—O3	178.6 (3)
C11—N2—C12—C1	178.6 (2)	C35—C36—N10—O3	−2.9 (5)
Ni1—N2—C12—C1	−4.7 (3)	C2—C3—C4—C5	1.0 (5)
C23—N4—C24—C20	−1.0 (4)	C1—C5—C4—C3	−1.0 (5)
Ni1—N4—C24—C20	178.6 (2)	C6—C5—C4—C3	178.2 (3)
C23—N4—C24—C13	178.6 (3)	C5—C6—C7—C8	−1.5 (6)
Ni1—N4—C24—C13	−1.8 (3)	C9—C8—C7—C6	−179.9 (4)
C14—N3—C13—C17	1.2 (4)	C12—C8—C7—C6	−1.2 (5)
Ni1—N3—C13—C17	−179.5 (2)	C20—C19—C18—C17	0.7 (6)
C14—N3—C13—C24	−178.3 (3)	C16—C17—C18—C19	−179.8 (4)
Ni1—N3—C13—C24	0.9 (3)	C13—C17—C18—C19	0.4 (6)
N4—C24—C13—N3	0.6 (4)	N3—C14—C15—C16	1.1 (6)
C20—C24—C13—N3	−179.8 (3)	C14—C15—C16—C17	−0.3 (6)
N4—C24—C13—C17	−179.0 (3)	C13—C17—C16—C15	0.0 (6)
C20—C24—C13—C17	0.6 (4)	C18—C17—C16—C15	−179.9 (4)
C12—N2—C11—C10	−0.5 (4)	C26—N6—C25—N5	177 (100)
Ni1—N2—C11—C10	−176.6 (2)	C25—N6—C26—C31	6.4 (5)
N4—C24—C20—C21	1.1 (4)	C25—N6—C26—C27	−172.4 (3)
C13—C24—C20—C21	−178.5 (3)	N6—C26—C31—C30	−177.7 (3)
N4—C24—C20—C19	180.0 (3)	C27—C26—C31—C30	1.1 (5)
C13—C24—C20—C19	0.4 (4)	C26—C31—C30—C29	−0.4 (5)
C4—C5—C6—C7	−177.4 (4)	C31—C30—C29—C28	−0.2 (6)
C1—C5—C6—C7	1.8 (5)	C31—C30—C29—N7	−178.1 (4)
C2—N1—C1—C5	0.9 (4)	N6—C26—C27—C28	177.7 (4)
Ni1—N1—C1—C5	−170.8 (2)	C31—C26—C27—C28	−1.2 (6)
C2—N1—C1—C12	−178.9 (3)	C28—C29—N7—O1	7.7 (8)
Ni1—N1—C1—C12	9.5 (3)	C30—C29—N7—O1	−174.4 (6)
C4—C5—C1—N1	0.1 (4)	C28—C29—N7—O2	−172.6 (5)
C6—C5—C1—N1	−179.2 (3)	C30—C29—N7—O2	5.3 (7)
C4—C5—C1—C12	179.8 (3)	C26—C27—C28—C29	0.6 (7)
C6—C5—C1—C12	0.6 (4)	C30—C29—C28—C27	0.1 (7)
N2—C12—C1—N1	−3.2 (4)	N7—C29—C28—C27	178.0 (4)
C8—C12—C1—N1	176.5 (3)		

### Hydrogen-bond geometry (Å, º)

**Table:**

*D*—H···*A*	*D*—H	H···*A*	*D*···*A*	*D*—H···*A*
O5—H5*A*···N9^i^	0.91 (3)	1.98 (4)	2.883 (4)	174 (5)
C39—H39*B*···O4^ii^	0.96	2.55	3.435 (7)	153
C22—H22···O1^iii^	0.93	2.57	3.469 (6)	162
C16—H16···O2^iv^	0.93	2.45	3.312 (6)	154

Symmetry codes: (i) *x*−1, *y*, *z*; (ii) −*x*+1, −*y*, −*z*+1; (iii) −*x*+2, −*y*+1, −*z*+2; (iv) *x*−1, *y*−1, *z*.
